# Over-expression of RRM2 predicts adverse prognosis correlated with immune infiltrates: A potential biomarker for hepatocellular carcinoma

**DOI:** 10.3389/fonc.2023.1144269

**Published:** 2023-03-28

**Authors:** Zhongqiang Qin, Bo Xie, Jingyu Qian, Xiang Ma, Lan Zhang, Jianzhu Wei, Zhaoying Wang, Longfei Fan, Ziyi Zhu, Zhen Qian, Hongxiang Yin, Fangquan Zhu, Yulin Tan

**Affiliations:** ^1^ Department of Interventional Radiology, The First Affiliated Hospital of Bengbu Medical College, Bengbu, China; ^2^ Department of Hepatobiliary Surgery, The First Affiliated Hospital of Bengbu Medical College, Bengbu, China; ^3^ Department of Surgical Oncology, Lu’an First People’s Hospital, Lu’an, China

**Keywords:** ribonucleotide reductase regulatory subunit M2, hepatocellular carcinoma, bioinformatics analysis, biomarker, The Cancer Genome Atlas

## Abstract

**Background:**

Ribonucleotide reductase regulatory subunit M2 (RRM2) has been reported to be an oncogene in some malignant tumors, such as lung adenocarcinoma, oral squamous cell carcinoma, glioblastoma, and breast cancer. However, the clinical significance of RRM2 in hepatocellular carcinoma has been less studied. The aim of this study was to assess the importance of RRM2 in hepatocellular carcinoma (HCC) based on the Cancer Genome Atlas (TCGA) database.

**Methods:**

The RRM2 expression levels and clinical features were downloaded from the TCGA database. Immunohistochemistry results between tumor tissues and normal tissues were downloaded from the Proteinatlas database. Meanwhile, the expression levels of RRM2 in tumor and paraneoplastic tissues were further verified by qRT-PCR and Western Blotting. Gene Ontology (GO)/Kyoto Encyclopedia of Genes and Genomes (KEGG) and protein-protein-interactions (PPI) network were constructed to analyze RRM2-related downstream molecules. In addition, RRM2 expression-related pathways performed by gene set enrichment analysis (GSEA). Association analysis of RRM2 gene expression and immune infiltration was performed by single-sample GSEA (ssGSEA).

**Results:**

The RRM2 expression level in tumor tissues was higher than normal tissues (*P <*0.001). The elevated expression of RRM2 in HCC was significantly correlated with T stage (*P <*0.05), pathologic stage (*P <*0.05), tumor status (*P <*0.05), histologic grade (*P<*0.001), and AFP (*P <*0.001). HCC with higher RRM2 expression was positively associated with worse OS (overall survival), PFS (progression-free survival), and DSS (disease-specific survival). In the univariate analysis, the expression of RRM2, T stage, M stage, pathologic stage, and tumor status were negatively correlated with OS (*P <*0.05). Further analysis using multivariate Cox regression showed that tumor status (*P<*0.01) and RRM2 expression (*P<*0.05) were independent prognostic factors of OS in HCC. GO/KEGG analysis showed that the critical biological process (chromosome condensation and p53 signaling pathway) might be the possible function mechanism in promoting HCC. Moreover, GSEA showed that several pathways were enriched in RRM2 high-expression samples, including PD-1 signaling, cell cycle, P27 pathway, and T cell receptor signaling pathway. RRM2 was significantly correlated with the infiltration level of CD8 T cells, Cytotoxic cells, DCs, Neutrophils, NK cells, and T helper cells (*P <*0.05).

**Conclusion:**

Over-expression of RRM2 predict adverse prognosis and is correlated with immune infiltrates in HCC. RRM2 may be a significant molecular biomarker for HCC diagnosis and prognosis.

## 1 Introduction

Liver cancer is the fourth leading cause of cancer-related mortality globally, and its incidence is rising. There are approximately 841,000 new cases of liver cancer and 782,000 deaths due to liver cancer each year (1). As the most common histologic type of liver cancer, hepatocellular carcinoma (HCC) is responsible for the majority of liver cancer morbidity and death (2). The combination of the anatomic extent of the tumor and the extent of the underlying liver disease has led to a variety of staging and scoring systems (3). Because the diversity of tumor stages and the functional reserve of the liver affects treatment, it may lead to significant differences in the prognosis of patients with the same cancer stage.

Among several staging systems, the American Joint Committee on Cancer (AJCC) staging system has been used to describe the purely anatomical extent of the disease. The TNM (tumor‐node‐metastasis) staging system includes the size and extent of the tumor (T stage), the number of nearby positive lymph nodes (N stage) and the presence of metastases (M stage). As the understanding of tumors increases, the staging system needs to be continuously. The latest TNM staging manual was also released in 2017 to accommodate the development of precision medicine (4, 5). For example, the original stage T1 tumor (single tumor without vascular invasion) was divided into T1a (single tumor ≤2 cm in diameter) and T1b (single tumor >2 cm in diameter without vascular invasion), while stage T2 was correspondingly adjusted from single tumor with vascular invasion to single tumor >2 cm in diameter with vascular invasion.

The treatment strategies for HCC include surgical resection, interventional therapy, chemotherapy, radiation therapy, and more recently, immunotherapy. The best/most promising approach to treating HCC still relies on surgical resection (6). However, only 10-30% of HCC patients have an opportunity to be treated with surgery, because of their active viral infection, poor liver function, severe liver cirrhosis, insufficient residual liver volume, or poor physical status (7). Even for those patients who accept surgery, the recurrence rate is as high as 70% in the next five years (8). Thus, the treatment outcomes for HCC remain unsatisfactory yet, especially for patients with advanced HCC (9).

Many factors are involved in the tumorigenesis and development of tumor, such as tumor microenvironment (TME) and immune escape (10). Immune cells induce tumor cells to death, and tumor cells can inactivate immune cells. The interaction can be phased into elimination, balance, and escape. In addition, immunotherapy, as an emerging treatment, has made significant progress based on fundamental insights into the molecular mechanisms of liver tumorigenesis (11). Immunotherapy has evolved from a systemic, nonspecific simulated immune system to a more targeted activation of specific parts of the immune system (12). Therefore, a better understanding of TME and the specific markers of HCC used for diagnosis and prognosis would provide substantial benefits for the treatment of the disease.

Ribonucleotide reductase (RR) is a structural unit required for DNA replication and repair, including RRM1 and RRM2. RRM1 shows relatively constant expression throughout the whole life of the cell, whereas RRM2 protein expression dynamically changes upon stimulation. RRM2 is essential for DNA synthesis and repair by producing dNTPs (13). RRM2 is also regarded as a vital component in tumor progression, a regulator of some oncogenes, and a promising tumor biomarker for many cancers, such as lung adenocarcinoma (14), oral squamous cell carcinoma (15), glioblastoma (16), and breast cancer (17). However, the potential use of RRM2 as a biomarker to diagnose HCC remains unclear. Therefore, we investigated the expression level of RRM2 in tumor and normal liver tissues based on The Cancer Genome Atlas (TCGA) database, and through bioinformatics analysis to verify whether RRM2 could be used as a meaningful biomarker in HCC.

## 2 Materials and methods

### 2.1 Collection and analysis of data

A total of 424 RNAseq data and corresponding clinical information of patients with HCC downloaded from the Cancer Genome Atlas (TCGA) database. Those 374 RNAseq data with clinical information converted into TPM (transcripts per million reads) format for further analysis. Unavailable and unclear clinical information is considered missing value. Based on the expression of RRM2 in tumor samples, they were divided into low expression and high expression groups, and differentially expressed genes were analyzed by Htseq Counts using the DESeq2 package. The log-fold change (log Fc) >2 and the adjusted *P* value <0.05 were set as the thresholds for a statistical difference. The normal liver samples were obtained from the GTEx (Genotype-Tissue Expression) database. The results of immunohistochemistry for tumor tissues and normal tissues downloaded from the HPA (Human Protein Atlas) database.

### 2.2 Real-time quantitative PCR analysis

Five pairs of fresh specimens were collected, confirmed as hepatocellular carcinoma by pathology or immunohistochemistry, for testing the levels of mRNA and protein of RRM2 from The First Affiliated Hospital of Bengbu Medical College. TRIzol ™ Reagent (Invitrogen, Cat No: 15596026) was used, and the total RNA of tissues samples was extracted. Then, the RT-qPCR performed using an ABScript II One-Step SYBR Green RT-qPCR Kit (Abclonal Cat: RK20404). The RRM2 primer sequence is as follow: forward 5’-GTGGAGCGATTTAGCCAAGAA-3’ and reverse 5’-CACAAGGCATCGTTTCAATGG-3’. GAPDH: forward 5’-CAGGAGGCATTGCTGATGAT-3’ and reverse 5’-GAAGGCTGGGGCTCATTT-3’. The 2-ΔCT value was used to present the target gene mRNA level of RRM2.

### 2.3 Western blotting

Protease Inhibitor Cocktail (APExBIO, Cat No: K1007) was used to lyse tissue samples in a radioimmunoprecipitation analysis (RIPA) Lysis Buffer (CWBIO, Cat: CW2333S). Then, the protein concentration measured by the BCA Protein Assay Kit (Beyotime, Cat No. P0012). The following antibodies were used: anti-RRM2 (CST, Cat: 65939T), anti-GAPDH (CST, Cat: 5174T), and anti-Rabbit IgG (Abclonal, Cat: AS014).

### 2.4 GO/KEGG analysis and PPI network construction

GO/KEGG analysis performed by Bioconductor package “clusterProfiler” (18). Screening of single-gene correlations related to RRM2 by R package (v3.6.3) from the TCGA-HCC database, the Pearson correlation value (≥0.5 or ≤-0.3, and *P*-value*<*0.001) and the z-test used to analyze the correlation between the level of RRM2 expression and its co-expression genes. STRING database and Cytoscape (v3.9.1) were used to build the PPI network (19).

### 2.5 Gene set enrichment analysis (GSEA)

GSEA (20) is a computational method based on the entire gene expression matrix. In this study, GSEA generated an ordered list of all genes according to their correlation with RRM2 expression, and the gene set permutations performed 1000 times. The expression profiles of 424 samples were input into GSEA. C2.cp.v7.2.symbols.gmt was selected as the reference gene set. The threshold value of GSEA for statistical significance was set as *P <*0.05 and FDR <0.25 after correlation. The adjusted *P* value and normalized enrichment score (NES) used to sort the pathways enriched in each phenotype. The ClusterProfiler version 3.14 package (18) used to analyze the GSEA enrichment and visualization.

### 2.6 Immune infiltration analysis

The marker gene of 24 immune cells extracted from the study of Bindea G’s research (21). The infiltration of 24 immune cell types in the tumor was analyzed using the single-sample GSEA (ssGSEA) method (22). The Spearman correlation between RRM2 and the 24 types of immune cells mentioned above, and for the analysis of immune cell infiltration between RRM2 high-expression and low-expression groups.

### 2.7 Statistical analysis

All statistical analyses performed in R (v3.6.3). The Wilcoxon rank-sum test was used for unpaired samples, while the Wilcoxon signed-rank test used for paired samples. The receiver operating characteristic (ROC) curve used to analyze whether RRM2 expression could be used as the diagnostic marker. Kruskal-Wallis test, Wilcoxon signed rank test, and logistic regression method used to analyze the correlation between clinicopathological characteristics and RRM2 expression. The chi-square test or Fisher exact was used to analyze the correlation between RRM2 expression and clinicopathological features. In addition, to assess the role of RRM2 expression in prognosis, we used the Kaplan-Meier method and Cox regression. In Cox regression analysis, variables with *P <*0.1 in univariate Cox regression were incorporated into multivariate Cox regression, and *P <*0.05 was considered statistically significant.

## 3 Results

### 3.1 Association between RRM2 expression and clinical characteristics

**Figure 1:**
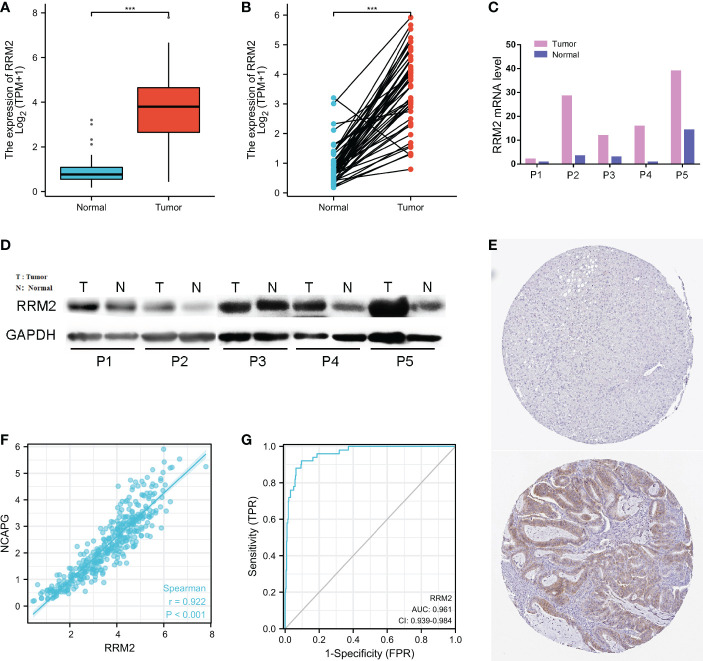
Relationship between RRM2 expression and hepatocellular carcinoma (HCC). **(A)** Differential expression of RRM2 between tumor tissues and normal tissues. **(B)** Differential expression of RRM2 between tumor tissues and matched para-cancerous tissues. **(C)** The mRNA level of RRM2. **(D)** The protein level of RRM2 in 5 paired of HCC tissues and adjacent tissues using Western blotting. **(E)** The results of Immunohistochemistry between the normal tissues and the tumor tissues. **(F)** The correlation analysis of mRNA expression levels between RRM2 and NCAPG was performed. **(G)** Diagnostic value of RRM2 expression in HCC. *** represents *P <*0.001.

The expression of RRM2 in hepatocellular carcinoma and normal tissues was analyzed, and the difference in RRM2 expression levels was confirmed (*P <*0.001, **Figures 1A, B**); RRM2 expression was higher in LIHC (Liver Hepatocellular Carcinoma) tissues than in normal tissues (*P <*0.001, **Figure 1A**). The expression levels of RRM2 in LIHC tissues and paired adjacent non-tumor tissues were analyzed. The results also confirmed that RRM2 was highly expressed in the tumor tissues (*P <*0.001, **Figure 1B**). The results of qRT-PCR and Western blotting showed that the mRNA and protein levels were higher in tumor tissues than in paired neighboring tissues (**Figures 1C, D**). Searching immunohistochemical results from the Proteinatlas database for tumor tissues and normal tissues, we found more protein expression in tumor tissues (**Figure 1E**). Interestingly, we found that RRM2 expression was positively correlated with non-structural maintenance of chromosomes condensin I complex subunit G (NCAPG), which promotes tumor development (*P <*0.001, **Figure 1F**) (23). In addition, ROC curves were used to analyze the diagnostic value of RRM2. The area under the curve (AUC) of RRM2 was 0.961, and the results suggest that RRM2 may be a potential diagnostic biomarker (**Figure 1G**).

**Figure 2:**
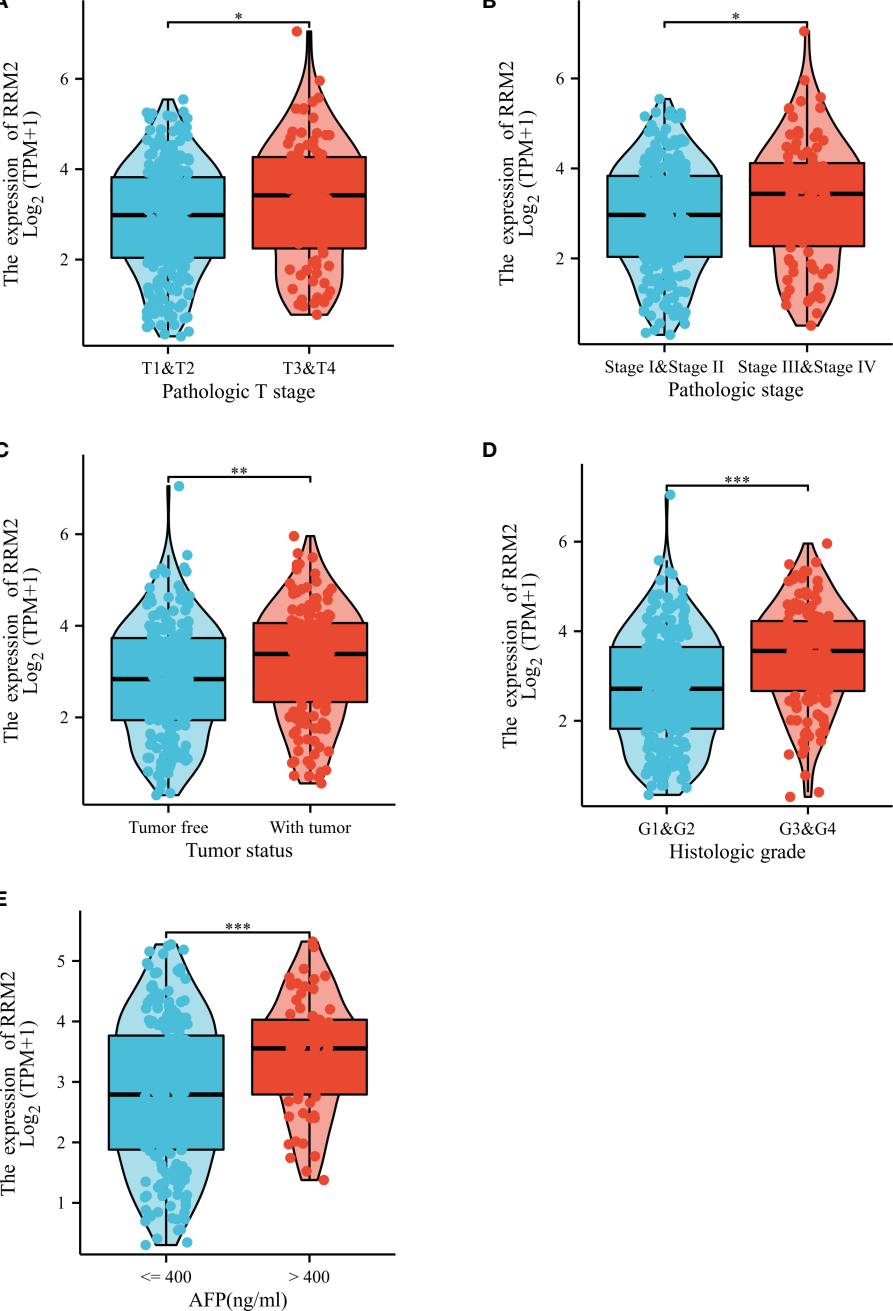
Correlation between RRM2 expression and clinical characteristics. Relationship of RRM2 expression with **(A)** T stage, **(B)** Pathologic stage, **(C)** Tumor status, **(D)** Histologic grade, and **(E)** AFP level. *represents *P <*0.05, ** represents *P <*0.01, *** represents *P <*0.001.

In addition, the Kruskal-Wallis test and Wilcoxon signed rank test used to analyze the correlation between clinical characteristics and RRM2 expression. The increased level of RRM2 expression were positively correlated with a higher grade of T stage (*P <*0.05, **Figure 2A**), Pathologic stage (*P <*0.05, **Figure 2B**), tumor status (*P <*0.01, **Figure 2C**), histologic grade (*P <*0.001, **Figure 2D**), and AFP levels (*P <*0.001, **Figure 2E**). Meanwhile, consistent results were also found using the chi-square test or Fisher exact test (**Table 1**). Furthermore, univariate logistic regression of RRM2 expression (**Table 2**) revealed that RRM2 expression was also closely related to clinical characteristics, including T stage (OR=1.698, 95% confidence interval (CI): 1.057-2.753, *P*=0.030), pathologic stage (OR=1.723, 95% confidence interval (CI): 1.063-2.821, *P*=0.029), tumor status (OR=1.640, 95% confidence interval (CI): 1.076-2.511, *P*=0.022), age (OR=0.629, 95% confidence interval (CI): 0.417-0.946, *P*=0.026), histologic grade (OR=2.893, 95% confidence interval (CI): 1.870-4.524, *P <*0.001), AFP level (OR=3.768, 95% confidence interval (CI): 2.084-7.065, *P <*0.001). Those results suggest that the expression of RRM2 may affect the progression of HCC.

**Table 1:** Association of RRM2 expression and clinical characteristics in HCC.

Characteristics	Low expression of RRM2	High expression of RRM2	*P*
n	187	187	
OS event, n (%)			0.023
Alive	133 (35.6%)	111 (29.7%)	
Dead	54 (14.4%)	76 (20.3%)	
T stage, n (%)			0.001
T1	110 (29.6%)	73 (19.7%)	
T2	37 (10%)	58 (15.6%)	
T3	32 (8.6%)	48 (12.9%)	
T4	5 (1.3%)	8 (2.2%)	
N stage, n (%)			0.629
N0	116 (45%)	138 (53.5%)	
N1	1 (0.4%)	3 (1.2%)	
M stage, n (%)			0.345
M0	125 (46%)	143 (52.6%)	
M1	3 (1.1%)	1 (0.4%)	
Pathologic stage, n (%)			< 0.001
Stage I	103 (29.4%)	70 (20%)	
Stage II	36 (10.3%)	51 (14.6%)	
Stage III	32 (9.1%)	53 (15.1%)	
Stage IV	4 (1.1%)	1 (0.3%)	
Tumor status, n (%)			0.029
Tumor free	112 (31.5%)	90 (25.4%)	
With tumor	66 (18.6%)	87 (24.5%)	
Gender, n (%)			0.269
Female	55 (14.7%)	66 (17.6%)	
Male	132 (35.3%)	121 (32.4%)	
Age, n (%)			0.034
≤60	78 (20.9%)	99 (26.5%)	
>60	109 (29.2%)	87 (23.3%)	
Weight, n (%)			0.103
≤70	85 (24.6%)	99 (28.6%)	
>70	90 (26%)	72 (20.8%)	
Height, n (%)			0.154
< 170	95 (27.9%)	106 (31.1%)	
≥170	78 (22.9%)	62 (18.2%)	
BMI, n (%)			0.946
≤25	89 (26.4%)	88 (26.1%)	
>25	82 (24.3%)	78 (23.1%)	
Residual tumor, n (%)			0.902
R0	167 (48.4%)	160 (46.4%)	
R1	8 (2.3%)	9 (2.6%)	
R2	1 (0.3%)	0 (0%)	
Histologic grade, n (%)			< 0.001
G1	41 (11.1%)	14 (3.8%)	
G2	98 (26.6%)	80 (21.7%)	
G3	44 (11.9%)	80 (21.7%)	
G4	2 (0.5%)	10 (2.7%)	
Adjacent hepatic tissue inflammation, n (%)			0.713
None	66 (27.8%)	52 (21.9%)	
Mild	51 (21.5%)	50 (21.1%)	
Severe	10 (4.2%)	8 (3.4%)	
AFP (ng/ml), n (%)			< 0.001
≤400	127 (45.4%)	88 (31.4%)	
>400	18 (6.4%)	47 (16.8%)	
Child-Pugh grade, n (%)			0.366
A	118 (49%)	101 (41.9%)	
B	9 (3.7%)	12 (5%)	
C	1 (0.4%)	0 (0%)	
Fibrosis. Ishak. score, n (%)			0.336
0	46 (21.4%)	29 (13.5%)	
1/2	14 (6.5%)	17 (7.9%)	
3/4	14 (6.5%)	14 (6.5%)	
5/6	40 (18.6%)	41 (19.1%)	
Vascular invasion, n (%)			0.188
No	114 (35.8%)	94 (29.6%)	
Yes	51 (16%)	59 (18.6%)	
Age, median (IQR)	64 (54.5, 69)	59 (51, 68)	0.019
Height, median (IQR)	168 (162, 175)	166 (160, 172)	0.041
Weight, median (IQR)	71 (61, 85)	69 (58, 79)	0.075
BMI, median (IQR)	24.39 (21.89, 29.04)	24.65 (21.32, 27.99)	0.457

OS, Overall Survival; IQR, interquartile range.

**Table 2:** Logistic regression analysis of RRM2 expression association with clinical pathological characteristics.

Characteristics	Total (N)	Odds Ratio (OR)	*P* value
T stage (T1&T2 vs. T3&T4)	371	1.698 (1.057-2.753)	0.030
N stage (N0 vs. N1)	258	2.522 (0.318-51.354)	0.426
M stage (M0 vs. M1)	272	0.291 (0.014-2.308)	0.288
Pathologic stage (Stage I &Stage II vs. Stage III &Stage IV)	350	1.723 (1.063-2.821)	0.029
Tumor status (With tumor vs. Tumor free)	355	1.640 (1.076-2.511)	0.022
Gender (Male vs. Female)	374	0.764 (0.494-1.179)	0.225
Age (>60 vs. ≤60)	373	0.629 (0.417-0.946)	0.026
Residual tumor (R0 vs. R1&R2)	345	1.044 (0.398-2.738)	0.930
Histologic grade (G1&G2 vs. G3&G4)	369	2.893 (1.870-4.524)	<0.001
AFP (ng/ml) (>400 vs. ≤400)	280	3.768 (2.084-7.065)	<0.001
Adjacent hepatic tissue inflammation (None vs. Mild &Severe)	237	1.207 (0.724-2.016)	0.471
Child-Pugh grade (A vs. B&C)	241	1.402 (0.581-3.452)	0.452
Vascular invasion (NO vs. YES)	318	1.403 (0.883-2.236)	0.152

### 3.2 Over expression of RRM2 could influence the prognosis of HCC patients

**Figure 3:**
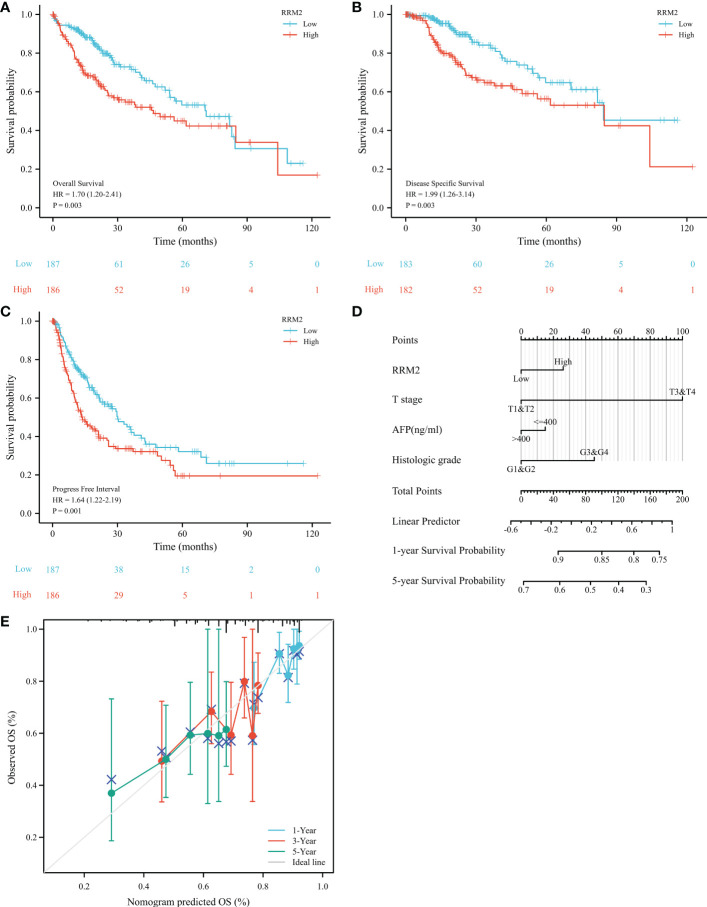
Increased expression of RRM2 indicated poor prognosis in HCC. Of the 374 cases, patients with high expression of RRM2 had significantly **(A)** shorter OS (*P*=0.003), **(B)** DSS (*P*=0.003), and **(C)** PFI (*P*=0.001); **(D)** A nomogram that integrate RRM2 and other prognostic factors in HCC from TCGA data; **(E)** The calibration curve of the nomogram.

The OS (Overall Survival) was significantly worse among patients with high RRM2 expression than in those patients with low expression (HR=1.70, 95% confidence interval (CI): 1.20-2.41, *P*=0.003, **Figure 3A**); The DSS (Disease-Specific Survival) (HR=1.99, 95% confidence interval (CI): 1.26-3.14, *P*=0.003, **Figure 3B**) and the PFI (Progress Free Interval) (HR=1.64, 95% confidence interval (CI): 1.22-2.19, *P*=0.001, **Figure 3C**) were significantly shorter in the high expression group than in the low expression group. In addition, we constructed a nomogram of OS to integrate RRM2 and other prognostic factors, like T stage, AFP levels, and histologic grade (**Figure 3D**). The calibration curve evaluated the nomogram’s performance of RRM2, and the C-index of OS was 0.649 (**Figure 3E**). In the univariate analysis, the T stage, M stage, pathologic stage, tumor status, and RRM2 expression level affected the prognosis of HCC patients (*P <*0.05). Further analysis by multivariate Cox regression showed that tumor status and RRM2 expression were independent prognostic risk factors of OS (HR=1.626, 95% confidence interval (CI): 1.010-2.619, *P*=0.045, **Table 3**) in HCC patients. Those results indicated that the expression level of RRM2 was associated with the prognosis of HCC patients.

**Table 3:** Univariate and multivariate Cox proportional hazards analysis for RRM2 expression.

Characteristics	Total (N)	Univariate analysis		Multivariate analysis	
		Hazard ratio (95% CI)	*P* value	Hazard ratio (95% CI)	*P* value
T stage	370				
T1&T2	277				
T3&T4	93	2.598 (1.826-3.697)	<0.001	1.658 (0.225-12.238)	0.620
N stage	258				
N0	254				
N1	4	2.029 (0.497-8.281)	0.324		
M stage	272				
M0	268				
M1	4	4.077 (1.281-12.973)	0.017	1.826 (0.415-8.028)	0.426
Gender	373				
Female	121				
Male	252	0.793 (0.557-1.130)	0.200		
Age	373				
≤60	177				
>60	196	1.205 (0.850-1.708)	0.295		
Pathologic stage	349				
Stage I &Stage II	259				
Stage III &Stage IV	90	2.504 (1.727-3.631)	<0.001	1.392 (0.189-10.257)	0.745
Histologic grade	368				
G1&G2	233				
G3&G4	135	1.091 (0.761-1.564)	0.636		
Vascular invasion	317				
No	208				
Yes	109	1.344 (0.887-2.035)	0.163		
Tumor status	354				
Tumor free	202				
With tumor	152	2.317 (1.590-3.376)	<0.001	1.867 (1.164-2.993)	0.010
Adjacent hepatic tissue inflammation	236				
None	118				
Mild &Severe	118	1.194 (0.734-1.942)	0.475		
AFP (ng/ml)	279				
≤400	215				
>400	64	1.075 (0.658-1.759)	0.772		
RRM2	373				
Low	187				
High	186	1.698 (1.197-2.409)	0.003	1.626 (1.010-2.619)	0.045

### 3.3 GO/KEGG and PPI network establishment for genes correlated with RRM2 in HCC

**Figure 4:**
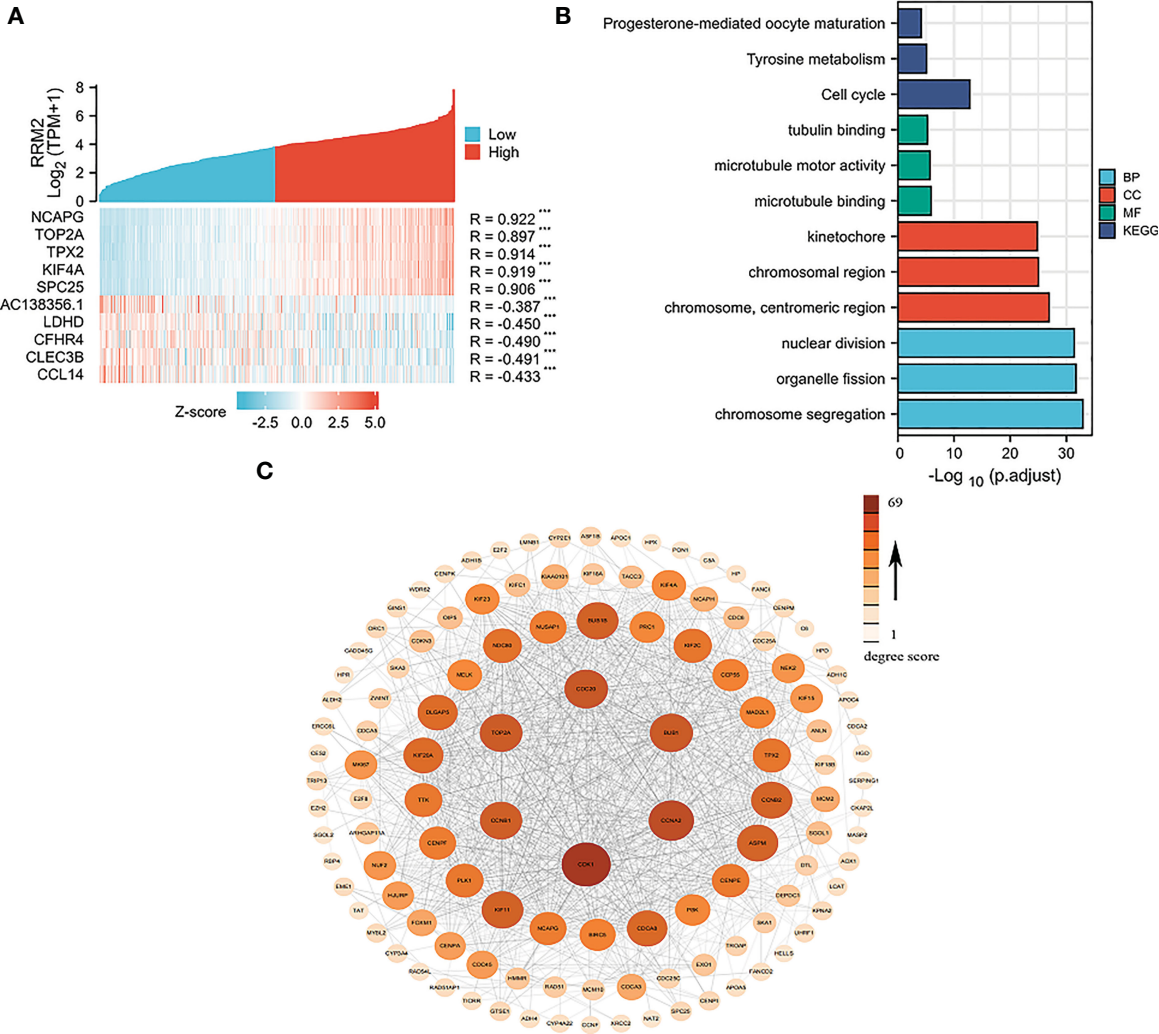
Network construction for RRM2 correlated genes in HCC. **(A)** Top 5 genes of positively or negatively correlated with RRM2 were shown in Heatmap. **(B)** GO analysis and KEGG pathway reveal the underlying mechanism of RRM2 in the promotion of HCC. **(C)** The PPI network of RRM2 interaction partners generated by STRING and Cytoscape. The color represents the degree score.

By single-gene correlation analysis, a total of 1814 genes correlated with RRM2 expression in TCGA-HCC patients (182 of 1814 genes were negatively correlated with RRM2 and 1632 genes were positively correlated with RRM2). The top five genes negatively or positively correlated with RRM2 shown in a Heatmap (**Figure 4A**). GO and KEGG analysis performed to understand the potential underlying mechanisms of RRM2 in the promotion of HCC. GO analysis showed that the critical biological process (chromosome segregation), cellular component (chromosome, centromeric region), and molecular functions (microtubule binding) may contribute to PLAU related biology. KEGG pathway analysis revealed that the cell cycle pathway, tyrosine metabolism, and progesterone-mediated oocyte maturation are significantly enriched by the RRM2 co-expressed genes (**Figure 4B**). Finally, the top 100 genes which were negatively or positively correlated with RRM2 were analyzed based on STRING and Cytoscape to construct the PPI network (**Figure 4C**).

### 3.4 GSEA show the RRM2-related pathways in HCC

**Figure 5:**
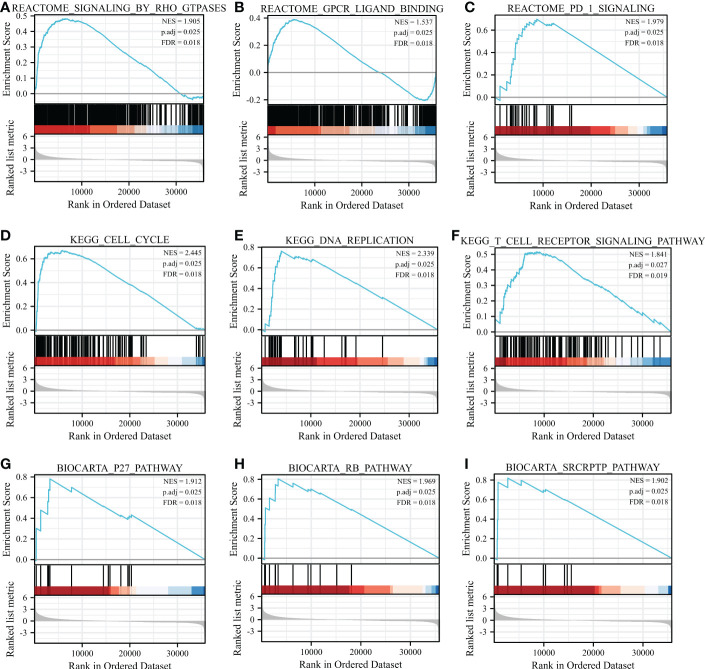
GSEA enrichment analysis results. GSEA results showed that **(A)** SIGNALING BY RHO FTPASES, **(B)** GPCR ligand binding, **(C)** PD1 signaling, **(D)** Cell cycle, **(E)** DNA replication, **(F)** T cell receptor signaling pathway, **(G)** P27 pathway, **(H)** RB pathway, **(I)** SRCRPTP pathway. FDR, false discovery rate; NES, normalized Enrichment Score.

Based on the normalized enrichment score (NES), we selected the most significant enrichment signaling pathway with high RRM2 gene expression (**Figure 5**; **Table 4**). GSEA analysis results showed that the increased expressed RRM2 phenotype was concentrated mainly in signaling by RHO, FTPASES (**Figure 5A**), GPCR ligand binding (**Figure 5B**), PD1 signaling (**Figure 5C**), cell cycle (**Figure 5D**), DNA replication (**Figure 5E**), T cell receptor signaling pathway (**Figure 5F**), P27 pathway (**Figure 5G**), RB pathway (**Figure 5H**), SRCRPTP pathway (**Figure 5I**).

**Table 4:** Results of gene set enrichment analysis (GSEA).

Description	Set size	Enrichment Score	NES	*P* value	q values	Rank
REACTOME_SIGNALING_BY_RHO_GTPASES	451	0.4816034	1.904913	0.0010070493	0.017521551	6513
REACTOME_GPCR_LIGAND_BINDING	458	0.3889179	1.536821	0.0010090817	0.017521551	5593
REACTOME_PD_1_SIGNALING	26	0.6983617	1.979295	0.0014245014	0.017521551	8685
KEGG_CELL_CYCLE	124	0.6711932	2.444768	0.0011235955	0.017521551	5787
KEGG_DNA_REPLICATION	36	0.7626604	2.339278	0.0013404826	0.017521551	3988
KEGG_T_CELL_RECEPTOR_SIGNALING_PATHWAY	108	0.5162139	1.841113	0.0023014960	0.018740623	8380
BIOCARTA_P27_PATHWAY	13	0.7812883	1.912125	0.0016051364	0.017521551	3226
BIOCARTA_RB_PATHWAY	13	0.8044410	1.968789	0.0016051364	0.017521551	3312
BIOCARTA_SRCRPTP_PATHWAY	11	0.8198548	1.901896	0.0016863406	0.017941945	2737

### 3.5 RRM2 expression is correlated with immune infiltration level in HCC

**Figure 6:**
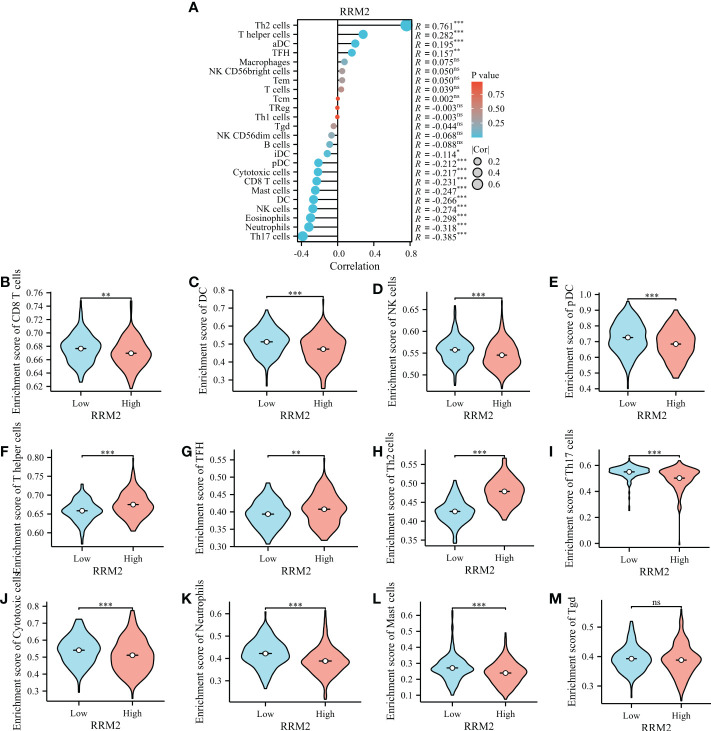
Relationship between RRM2 expression and immune infiltration. **(A)** Relationship between RRM2 expression and immune cells. **(B–M)** Further analysis in different immune cell subpopulations in the high- and low-expression groups of RRM2. *represents *P <*0.05, ** represents *P <*0.01, *** represents *P <*0.001. NS, Non Significance.

Immune infiltration levels are independent predictors of cancer survival. Therefore, we investigated whether the RRM2 expression is related to the level of immune infiltration in HCC. Through the evaluated correlation between RRM2 and 24 immune cell subsets in HCC, we found that RRM2 has a positive correlation with Th2 cells, T helper cells, and T follicular helper (TFH) cells; On the other hand, RRM2 has a negative relationship with Neutrophils, DC, CD8 T cells, and cytotoxic cells (**Figure 6A**). Further analysis showed significant difference in the RRM2 expression level among the infiltrating immune cells, including CD8 T cells, DC, NK cells, pDC, T helper cells, TFH, Th2 cells, Th17 cells, cytotoxic cells, neutrophils, mast cells, and Tgd (**Figures 6B–M**).

## 4 Discussion

Liver Cancer is the sixth most common cancer in the world and the fourth leading cause of cancer death. Hepatocellular carcinoma (HCC) is the most common pathological type. Therefore, we usually use HCC to refer to liver cancer in clinical work (1, 2). Although there are multiple strategies to treat this kind of malignant tumor, the outcomes are often unsatisfactory, especially in those advanced HCC patients, whose survival time is always no more than a year (24). This condition is caused by three key factors: lack of early diagnosis; the majority of HCC cases with a background of hepatitis and cirrhosis; and the tolerance of HCC to treatment (25). Thus, more attention should be given to early prediction and diagnosis. It’s encouraging that several biomarkers were found correlated with HCC recently, which can be helpful in HCC treatment.

RRM2 is a member of ribonucleotide reductase family, which has been proven to participate in the regulation and modification of proteins. In recent years, RRM2 has been defined as a vital component in tumor progression, as well as a promising tumor biomarker for many cancers. In lung adenocarcinoma, silencing RRM2 expression exerted anti-tumor effects by activating the cGAS/STING signaling pathway. In addition, it increased the infiltration of CD8+ T cells and cooperated with radiation to inhibit LUAD cell proliferation, promote apoptosis (26). Over-expression of RRM2 can promote breast cancer metastasis by activating PI3K/AKT signaling pathway to induce cell invasion, migration and vascular endothelial growth factor expression (27). However, RRM2 in HCC, one of the most common cancers, remained to be further elucidated.

Through the bioinformatics analysis, we found that RRM2 expression was increased in hepatocellular carcinoma compared with normal liver tissues, indicating a potential function for RRM2 in tumorigenesis. Increased RRM2 expression was correlated with poor survival of patients with HCC. The OS, DSS, and PFI were significantly worse among patients with high RRM2 expression than those with low expression.

In this study, we also found that RRM2 was upregulated in HCC with a higher clinicopathological grade, such as T stage, pathologic stage, tumor status, histologic grade, and AFP level. The results indicated that RRM2 expression could be used as survival classifiers for HCC stage evaluation. To further investigate the role of RRM2 in HCC, the data downloaded from TAGA was used for GSEA. The results indicated that the elevated expression of genes was hugely enriched in pathways which were related to tumorigenesis, such as PD1 signaling, P27 pathway, and T cell receptor signaling pathway. In conclusion, the expression level of RRM2 has a strongly correlates with the morbidity and prognosis of HCC.

Additionally, we evaluated the correlation between RRM2 expression and immune cell levels by filtering the transcriptomic data, which could overcome the limitation of calculation methods. This could help us depict the immune infiltration in tumors. We found that RRM2 expression was significantly negatively correlated with neutrophils, CD8 T cells, DCs, and cytotoxic cells, and that immune cells play a crucial role in cancer immunology. It is certain that most of the immune cells could play a positive impact on clinical outcomes. The above results proved that RRM2 may be correlated with the mechanism of immune infiltration and may play a crucial role in the occurrence of HCC.

Although the findings in our study showed the correlation between RRM2 and HCC, there were still some deficiencies. First of all, we only use one database for analysis, and the results should be cross-validated by multiple databases. Secondly, the results lack validation from *in vitro* and *in vivo* experiments, so we could not confirm its specific molecular mechanism. Therefore, we may ignore some crucial biological information in our study.

In summary, we firstly revealed the clinical value of RRM2 in HCC by bioinformatic analysis. Further functional assays must be performed to consolidate the results from the present work. Besides, a broader comparison between RRM2 and currently reported markers in HCC is beneficial to improve the biomarker research in the field.

## Data availability statement

The datasets presented in this study can be found in online repositories. The names of the repository/repositories and accession number(s) can be found below: https://www.ncbi.nlm.nih.gov/genbank/, https://www.ncbi.nlm.nih.gov/.

## Author contributions

YT, BX, and FZ designed the project. ZQQ, ZW, XM, HY, and LZ performed the experiments. JW, ZQ, and JQ discussed the results. ZQQ, LF, and ZZ wrote the manuscript. All authors contributed to the article and approved the submitted version.
